# Drug-Loaded Mesoporous Silica Nanoparticles Enhance Antitumor Immunotherapy by Regulating MDSCs

**DOI:** 10.3390/molecules29112436

**Published:** 2024-05-22

**Authors:** Changlin Xu, Nida Amna, Yuchen Shi, Rong Sun, Chenhui Weng, Jiaoyu Chen, Huaxing Dai, Chao Wang

**Affiliations:** Institute of Functional Nano & Soft Materials (FUNSOM), Jiangsu Key Laboratory for Carbon-Based Function Materials and Devices, Soochow University, Suzhou 215123, China; georgelinxu@163.com (C.X.); 20237114004@stu.suda.edu.cn (N.A.); 2114401011@stu.suda.edu.cn (Y.S.); rsun123@stu.suda.edu.cn (R.S.); ellenweng7@163.com (C.W.); 2014401056@stu.suda.edu.cn (J.C.)

**Keywords:** myeloid-derived suppressor cells, tumor therapy, stattic, PEG-MSN, ICB therapy

## Abstract

Myeloid-derived suppressor cells (MDSCs) are recognized as major immune suppressor cells in the tumor microenvironment that may inhibit immune checkpoint blockade (ICB) therapy. Here, we developed a Stattic-loaded mesoporous silica nanoparticle (PEG-MSN-Stattic) delivery system to tumor sites to reduce the number of MDSCs in tumors. This approach is able to significantly deplete intratumoral MSDCs and thereby increase the infiltration of T lymphocytes in tumors to enhance ICB therapy. Our approach may provide a drug delivery strategy for regulating the tumor microenvironment and enhancing cancer immunotherapy efficacy.

## 1. Introduction

Cancer, characterized by uncontrolled cell proliferation, tissue invasion, and metastatic spread to distant organs, poses a significant threat to human health [1,2]. In recent years, immune checkpoint blockade (ICB) therapy, which targets inhibitory receptors on immune cells, has revolutionized the clinical management of various malignancies. ICB therapy aims to restore the antitumor immune response by blocking the interaction between immune checkpoint proteins, such as programmed cell death protein 1 (PD-1) and its ligand (PD-L1), thereby enhancing the activity of T cells against tumor cells. Although ICB therapy has drastically changed the landscape of clinical cancer treatment, up to 50% of patients still have no objective response to ICB therapy [3,4,5]. The main reason is that tumors tend to shift the tumor immune microenvironment (TIME) into an immunosuppressive state against host immunity. The TIME is composed of various cellular and molecular components, including immune cells, cancer cells, fibroblasts, the extracellular matrix, and soluble factors, which interact in a coordinated manner to influence the overall immune response [6,7,8,9]. Tumors exhibit enrichment of pathways involved in evading immune surveillance, including those involved in enhancing negative immune regulatory pathways and recruiting immunosuppressive cells, leading to the suppression of immune cell functions and antitumor immune responses [10,11,12]. These mechanisms contribute to the suppression of immune cell functions and dampening of the antitumor immune response. One type of typical immunosuppressive cell is myeloid-derived suppressor cells (MDSCs), which are a highly heterogeneous population of immature myeloid cells with different transcriptional activities and differentiation states that exhibit immunosuppressive effects on T cells under pathological conditions. In addition, MDSCs are characterized by their plasticity, with diverse subsets exhibiting distinct transcriptional activities, differentiation states, and functional properties. These cells can be further classified into two main subsets: polymorphonuclear MDSCs (PMN-MDSCs) and monocytic MDSCs (M-MDSCs). PMN-MDSCs resemble neutrophils and are characterized by the expression of granulocyte markers, while M-MDSCs are more similar to monocytes and macrophages. MDSCs exert their immunosuppressive effects through various mechanisms, including the production of reactive oxygen species (ROS) and reactive nitrogen species (RNS), the secretion of immunosuppressive cytokines (IL-10 and TGF-β), and direct interactions with T cells, leading to T cell anergy or apoptosis [13,14,15]. Therefore, given the detrimental impact of MDSCs on the antitumor immune response, their elimination or functional modulation within the tumor microenvironment represents a promising strategy to enhance the efficiency of cancer immunotherapy.

Previous studies have shown that targeting MDSCs within tumors can help enhance the immune response within tumors and subsequently inhibit tumor growth and metastasis [16,17,18,19]. Typically, some small-molecule inhibitors can reduce MDSC viability and immunosuppressive function by inhibiting signal transducer and activator of transcription (STAT) signaling [20,21,22,23]. One such small-molecule inhibitor is Stattic, which targets the signal transducer and activator of transcription 3 (STAT3) protein. STAT3 is a member of the STAT protein family and plays a crucial role in tumor cell growth, angiogenesis, and the evasion of immune surveillance. Hyperactivation of STAT3 has been observed in various malignancies and is associated with tumorigenesis and immune tolerance [24,25]. Specifically, Stattic can selectively bind to the SH2-binding domain and inhibit the activation, dimerization, and translocation of STAT3, which in turn dampens its downstream oncogenic and immunosuppressive effects [26,27]. However, small-molecule drugs such as Stattic face several challenges in clinical application, including rapid metabolism in the body, short half-lives, and poor bioavailability. These limitations can result in suboptimal drug concentrations at the target sites, reduced therapeutic efficiency, and a narrow therapeutic window.

To address these challenges, nanoparticles have been explored as promising drug delivery systems to improve the pharmacokinetic properties and bioavailability of small-molecule drugs [28]. Mesoporous silica nanoparticles (MSNs) have become one of the most promising nanomaterials due to their good biosafety, high drug loading capacity, and easy modification [29,30]. MSNs can be functionalized with polyethylene glycol (PEG) to enhance their stability, circulation time, and biocompatibility while also reducing immunogenicity and nonspecific protein adsorption. Here, we developed a nanoparticle delivery system via the PEGylation of mesoporous silica nanoparticles (PEG-MSNs) loaded with the small-molecule inhibitor Stattic. We demonstrated that Stattic-loaded nanoparticles (PEG-MSNs-Stattic) can enhance antitumor immunotherapy efficacy by reducing the number of MDSCs within tumors. Through intratumoral injection, we found that PEG-MSNs-Stattic can remain in tumors for a long time and extend the half-life of small-molecule drugs through sustained drug release. We found that PEG-MSNs-Stattic can inhibit tumor growth by approximately 90% after combination with an immune checkpoint inhibitor (aPD-L1). Treatment with PEG-MSNs-Stattic not only decreased the number of MDSCs but also increased the proportion of CD4^+^ and CD8^+^ T cells within tumors, thereby dramatically enhancing the efficacy of ICB therapy.

## 2. Results

### 2.1. Characterization of PEG-MSNs and Their Toxicity, Drug Loading, and Release In Vitro

**Figure 1:**
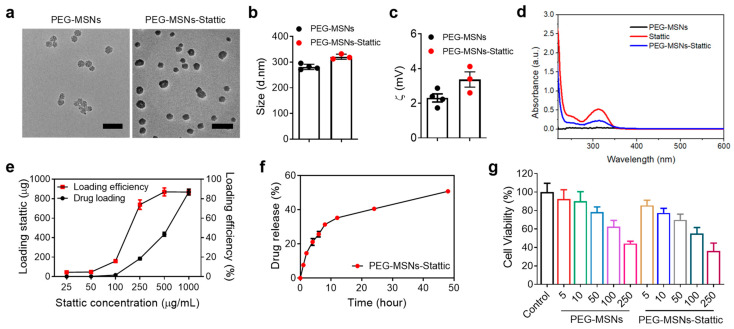
Characterization and drug loading and cytotoxicity evaluation. (**a**) Transmission electron microscopy (TEM) images of PEG-MSNs and PEG-MSNs-Stattic. Scale bar: 100 nm. (**b**) Average size and (**c**) zeta potential of PEG-MSNs and PEG-MSNs-Stattic measured by dynamic light scattering (DLS). (**d**) Characteristic absorption peak of PEG-MSNs, Stattic, and PEG-MSNs-Stattic. (**e**) Drug loading efficiency of Stattic. (**f**) Drug release of PEG-MSNs-Stattic in vitro. (**g**) Cytotoxicity of PEG-MSNs and PEG-MSNs-Stattic determined by the MTT assay. The data are shown as the mean ± SEM.

Mesoporous silica nanoparticles (MSNs) were synthesized according to our previously established protocols [31]. Since previous studies have shown that 5 kDa PEG modification can significantly increase the stability of nanoparticles while reducing protein adsorption on the nanoparticle surface [32,33], we modified MSNs with 5 kDa PEG to improve their stability and biocompatibility. PEG-MSNs-Stattic were prepared by mixing a PBS solution of PEG-MSNs with Stattic by shaking and centrifugation. Transmission electron microscopy (TEM) images showed that the sizes of both the PEG-MSNs and the PEG-MSNs-Stattic were approximately 30–50 nm (Figure 1a). The hydrodynamic size and zeta potential of the PEG-MSNs were approximately 281.4 ± 10.4 nm and 2.2 ± 0.2 mV, whereas those of the PEG-MSNs-Stattic were approximately 320 ± 12 nm and 3.6 ± 0.2 mV, respectively (Figure 1b,c). PEG modification can significantly improve the stability of MSNs in different solutions. Previous studies have shown that PEGylation can effectively reduce the nonspecific binding of MSNs to human serum albumin (HSA) and increase the stability of MSNs in aqueous or PBS solutions [34,35,36]. PEG-MSNs did not have a significant absorption peak, and Stattic exhibited a high absorption peak at approximately 320 nm. The absorption peak of PEG-MSNs-Stattic at approximately 320 nm suggested that Stattic was successfully loaded into the PEG-MSNs (Figure 1d).

The drug loading and release efficiency of PEG-MSNs-Stattic was evaluated by high-performance liquid chromatography (HPLC). As the concentration of Stattic increased, the drug loading increased exponentially. Notably, the drug loading efficiency increased with increasing drug concentration and reached saturation at approximately 85% when the drug concentration exceeded 500 μg/mL (Figure 1e). Approximately 50% of the Stattic was released from the PEG-MSNs within 50 h (Figure 1f). To evaluate the cytotoxicity of PEG-MSNs and PEG-MSNs-Stattic, different concentrations of nanoparticles were incubated with RAW264.7 cells for 24 h. Neither PEG-MSNs nor PEG-MSNs-Stattic had obvious cytotoxicity at concentrations less than 50 μg/mL (Figure 1g). These results indicated that PEG-MSNs can be used to effectively load the small-molecule inhibitor Stattic and release the drug slowly with good biosafety.

### 2.2. Biodistribution of PEG-MSNs in Tumors

**Figure 2:**
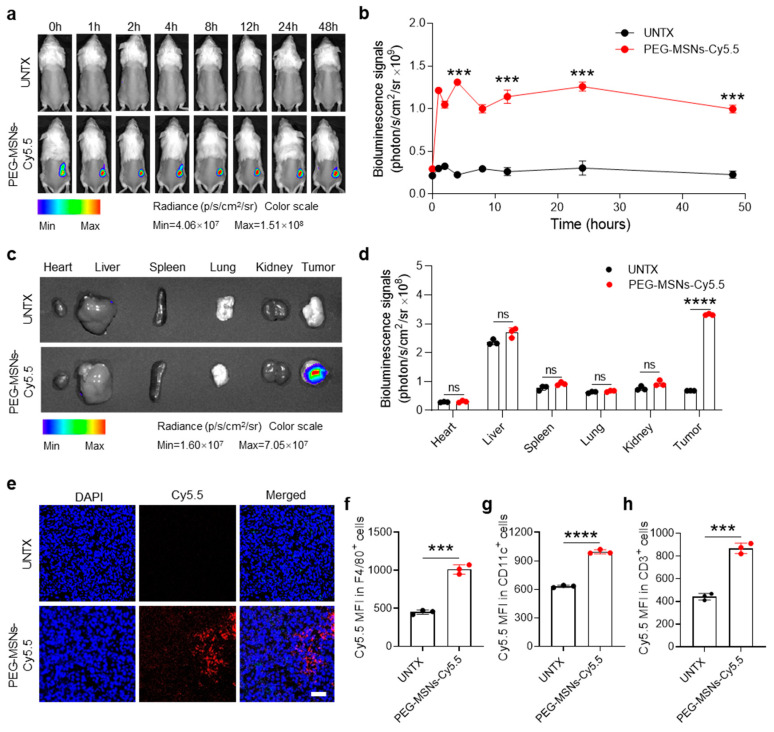
Biodistribution of PEG-MSNs. (**a**) In vivo bioluminescence imaging of the untreated and PEG-MSN-Cy5.5-injected mice from 0 h to 48 h and (**b**) corresponding quantification results. (**c**) Ex vivo imaging of major organs and tumors at 48 h and (**d**) corresponding quantification results. (**e**) Representative images of tumor immunofluorescence staining. Scale bar: 50 μm. (**f**–**h**) Flow cytometry analysis of the Cy5.5 mean fluorescence intensity (MFI) in F4/80^+^, CD11c^+^, and CD3^+^ cells. The data are shown as the mean ± SEM. Statistical analysis was performed using Student’s *t* test. *** *p* < 0.001; **** *p* < 0.0001; ns, nonsignificant.

To examine the retention and biodistribution of PEG-MSNs after intratumoral (i.t.) injection, we established a subcutaneous tumor model and conjugated PEG-MSNs with Cy5.5 dye. After intratumoral injection of PEG-MSNs-Cy5.5, the tumor maintained a strong Cy5.5 fluorescence signal within 48 h (Figure 2a,b). Ex vivo bioluminescence images revealed fluorescence signals in major organs (heart, liver, spleen, lung, and kidney) as well as in tumors at 48 h after injection. The results showed that, rather than in other major organs, the PEG-MSNs mainly accumulated in the tumor (Figure 2c,d). Representative confocal images further confirmed the retention of PEG-MSNs-Cy5.5 within tumors after injection for 48 h (Figure 2e). The above results indicated that PEG-MSNs can stay in tumors for a long time, thereby achieving sustained drug release and extending the drug half-life.

To explore whether PEG-MSNs can interact with immune cells in tumors, we analyzed the fluorescence signal intensity of Cy5.5 in F4/80^+^, CD11c^+^, and CD3^+^ cells after intratumoral injection of PEG-MSNs-Cy5.5 for 48 h. Flow cytometry analysis of Cy5.5 in different types of cells revealed that PEG-MSNs can interact with macrophages, dendritic cells, and T cells in tumors (Figure 2f,g). Together, our results suggested that PEG-MSNs can stay in tumors for a long time and be more readily captured and recognized by immune cells.

### 2.3. PEG-MSNs-Stattic in Combination with aPD-L1 Blockade Inhibited Tumor Growth

**Figure 3:**
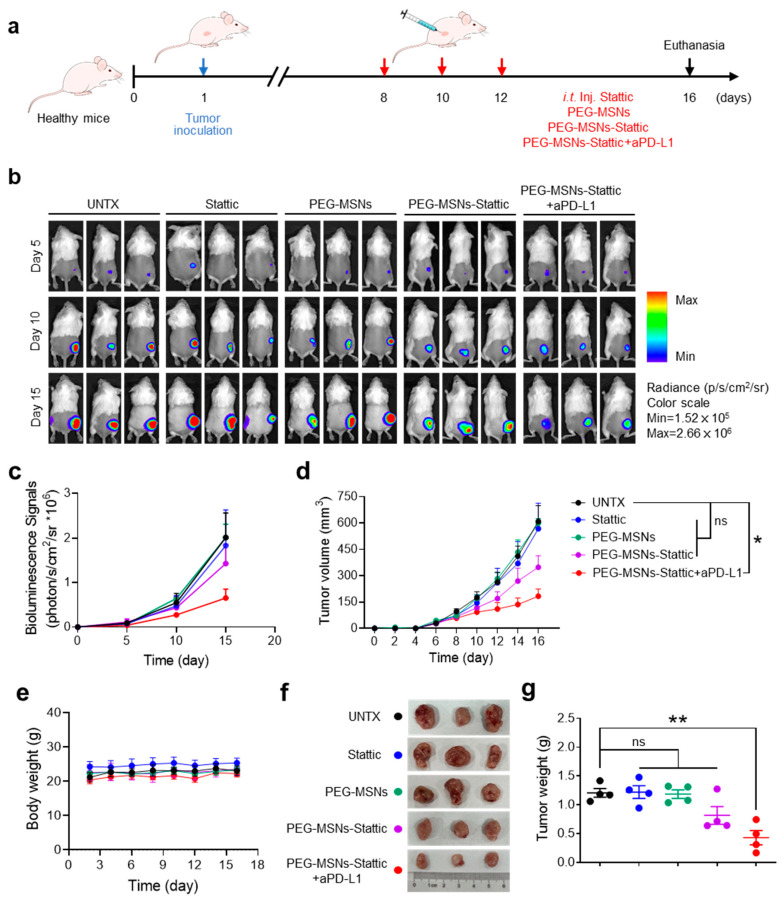
PEG-MSNs-Stattic enhanced the efficacy of anti-PD-L1 therapy. (**a**) Scheme of tumor-bearing mice injected with Stattic, PEG-MSNs, PEG-MSNs-Stattic, or PEG-MSNs-Stattic+aPD-L1. (**b**) In vivo bioluminescence imaging of CT26-Luc tumors. (**c**) Quantitative bioluminescence signals of tumors in the five groups. (**d**) Tumor growth curves of mice in different treatment groups. (**e**) Body weights of mice subjected to different treatments. (**f**) Representative tumor images and (**g**) quantification of the tumor weights. The data are shown as the mean ± SEM. Statistical analysis was performed using Student’s *t* test. * *p* < 0.05; ** *p* < 0.01; ns, nonsignificant.

We next questioned whether PEG-MSNs-Stattic could inhibit tumor growth and enhance ICB therapy by altering the tumor microenvironment. The tumor model was established by subcutaneous injection of CT26-Luc cells. To investigate the influence of different treatments on tumor growth, Stattic, PEG-MSNs, aPD-L1, or PEG-MSNs-Stattic were separately injected into tumor-bearing mice on days 8, 10, and 12 (Figure 3a). The results showed that intratumorally injected PEG-MSNs and Stattic had no obvious effect on tumor growth. As expected, tumor growth was obviously inhibited in the PEG-MSNs-Stattic-treated group, which exhibited excellent antitumor efficacy after combination with aPD-L1 blockade, and the body weights of the mice did not obviously change (Figure 3b–g). Our results also suggested that aPD-L1 treatment alone has no significant effect on tumor growth (Figure S1), which further indicated that PEG-MSNs-Stattic treatment may maximize the effect of aPD-L1 therapy by regulating the number of MDSCs within tumors.

### 2.4. PEG-MSNs-Stattic Enhanced ICB Therapy by Reducing MDSC Numbers in Tumors

**Figure 4:**
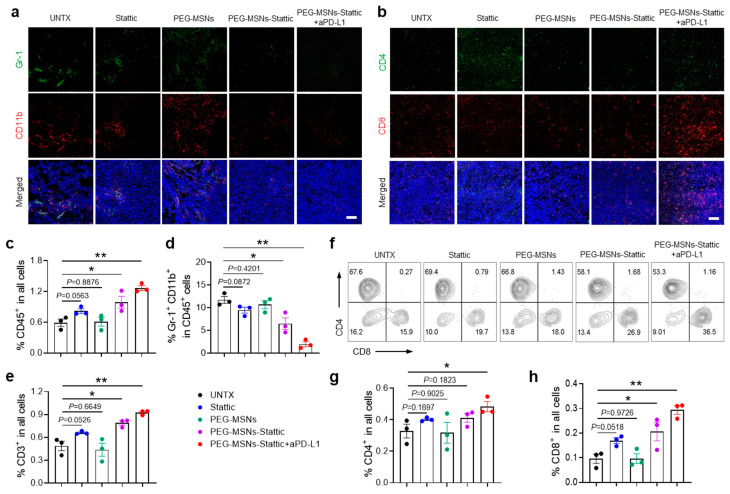
PEG-MSNs-Stattic enhanced ICB therapy by reducing the number of MDSCs within tumors. (**a**) Representative immunofluorescence images of MDSCs (Gr-1^+^CD11b^+^). Scale bars, 100 μm. (**b**) Representative immunofluorescence images of T cells within tumors (CD4^+^ and CD8^+^). Scale bars, 100 μm. (**c**–**e**) Corresponding quantitative results for CD45^+^, CD11b^+^Gr-1^+^, and CD3^+^ cells. (**f**–**h**) Representative flow cytometry plots for CD4^+^ and CD8^+^ T cells and the corresponding quantitative results. The data are shown as the mean ± SEM. Statistical analysis was performed using Student’s *t* test. * *p* < 0.05; ** *p* < 0.01.

According to previous research, Stattic effectively inhibits the activation of STAT3 [37], which reduces the immunosuppressive effect of MDSCs [38]. Unlike other treatment strategies [39], we did not directly target and kill tumor cells but focused on regulating the tumor immunosuppressive microenvironment, which showed synergetic therapeutic efficiency after combination with the immune checkpoint inhibitor. The low efficiency of PEG-MSNs was consistent with the findings of previous studies showing that the use of PEG-MSNs alone is not sufficient for exerting effective antitumor effects [40,41]. To explore the changes in the tumor immune microenvironment after different treatments, we further analyzed the numbers of immune cells and MDSCs in tumors through immunofluorescence and flow cytometric analysis. Comparative quantitative imaging of tumors demonstrated that the area of MDSCs dramatically decreased in PEG-MSNs-Stattic+aPD-L1-treated mice, while the areas of CD4^+^ and CD8^+^ T cells increased significantly (Figure 4a,b). Flow cytometry analysis revealed a large increase in lymphocytes (CD45^+^) after PEG-MSNs-Stattic+aPD-L1 treatment (Figure 4c). However, further analysis revealed that the proportion of MDSCs decreased significantly (Figure 4d and Figure S2). In comparison, the numbers of CD3^+^, CD3^+^CD4^+^, and CD3^+^CD8^+^ T cells increased significantly in the tumors after PEG-MSNs-Stattic+aPD-L1 treatment (Figure 4e–h and Figure S3). In addition, there was no obvious change in the PEG-MSNs and Stattic treatment groups, while the PEG-MSNs-Stattic treatment group had a reduced number of MDSCs in the tumors. Therefore, the above results indicated that PEG-MSNs-Stattic can decrease the number of MDSCs in tumors, subsequently inducing robust antitumor immunity after synergizing with aPD-L1 blockade.

## 3. Materials and Methods

### 3.1. Materials

Hexadecyl cetyltrimethylammonium bromide (CTAB), 3-aminopropyltriethoxysilane (APTES), N-hydroxysuccinimide (NHS), and 1-ethyl-3-(3-dimethyl-aminopropyl) carbodiimide (EDC) were obtained from Aladdin (Los Angeles, CA, USA). Tetraethyl orthosilicate (TEOS) was obtained from Beijing BaiLingWei Technology Co., Ltd. (Beijing, China). Triethanolamine (TEA) and ethanol were purchased from Sigma Aldrich (St. Louis, MO, USA). MethoxyPEG carboxyl groups (mPEG-CH_2_CH_2_COOH, 5K) were purchased from ACMEC (Shanghai, China). Deionized water was used in all the experiments. The antibodies and cell lines used in this study are shown in Table 1.

### 3.2. Mice and Cell Lines

BALB/c male and female mice (6–8 weeks, ~20 g) were purchased from the Experimental Animals Center of Soochow University. All mice were kept under a 12 h light–dark cycle (8:00–20:00 light; 20:00–8:00 dark) with a constant room temperature (21 ± 2 °C) and relative humidity (40–70%). All mice had free access to food and water. The maximum tumor load allowed was 1000 mm^3^, and the mice were euthanized at the time of the final experiment. The experimental methods were approved by the animal welfare authorities after weighing their statistical power, feasibility, and ethics. All our experiments were approved by the Animal Protection and Use Committee and were carried out under ethical standards (No. SUDA20210201A03).

All cell lines were obtained from the Shanghai Cell Bank of the Chinese Academy of Sciences. CT26-Luc (catalog: TCM37) cells were cultured in Roswell Park Memorial Institute (RPMI, HyClone. Logan, UT, USA) 1640 medium supplemented with 10% FBS, 1% penicillin, and streptomycin at 37 °C in 5% CO_2_.

### 3.3. Preparation and Characterization of PEG-MSNs and PEG-MSNs-Stattic

A mixture containing 320 mg of TEA and 2 g of CTAB was stirred at a rate of 500 rpm for 30 min, and 6 mL of TEOS was added during the stirring process. After 45 min, the precipitate was collected after centrifugation at 15,500 rpm for 45 min. The precipitate was subsequently resolved with 240 mL of absolute ethanol and 9 mL of hydrochloric acid. The mixture was boiled for 24 h to remove the excess CTAB and washed with ethanol. The resulting pellet was dried to obtain nanoparticles with no modification. To improve their biocompatibility and stability, the synthesized particles were subjected to a surface functionalization process involving the use of polyethylene glycol. After dispersing them in absolute ethanol, the nanoparticles were modified by adding APTES after 12 h of refluxing. The product was collected and washed with water 3 times. HEPES buffer was used to disperse mPEG-CH_2_CH_2_COOH, and the carboxyl group was activated with NHS and EDC. The particles were stirred in the solution at room temperature for approximately 24 h. Then, the mixture was subjected to centrifugation at 15,500 rpm for 45 min. The mixture was dried to obtain PEG-MSNs. Then, 500 ng of Stattic was added to 600 µL of a PBS solution of PEG-MSNs (2 mg/mL). The solution was mixed through vigorous shaking, left to stand for 0.5 h, and vortexed for 0.5 h. The precipitate was collected by centrifugation at 12,000 rpm for 10 min and dried to obtain PEG-MSNs-Stattic.

To observe the size and dispersibility of the particles, PEG-MSNs and PEG-MSNs-Stattic were evaluated with an FEI TF20 field emission transmission electron microscope. PEG-MSNs and PEG-MSNs-Stattic were dispersed in water and ultrasonically dispersed evenly. The carbon-coated 400-mesh grid was activated first, after which a small amount of liquid was added to the grids. The resulting materials were detected by a UV spectrophotometer. Finally, the PEG-MSNs and PEG-MSNs-Stattic were observed via TEM after drying. Then, the PEG-MSNs and PEG-MSNs-Stattic were dispersed in water, and the zeta potential (Nano ZS90, Malvern, Worcestershire, England) was used for dynamic light scattering analysis to determine the hydrodynamic size and zeta potential.

### 3.4. Drug Loading and Drug Release Evaluation

The drug loading efficiency of PEG-MSNs was studied by loading different concentrations of the chemotherapeutic drug Stattic onto the nanoparticles. A series of solutions containing varying amounts of Stattic (0, 25, 50, 100, 250, 500, or 1000 µg) were prepared in 600 µL of a 2 mg/mL PBS solution of PEG-MSNs. The mixtures were then mixed with vigorous shaking to ensure a uniform distribution of the drug molecules. Then, the mixtures were allowed to stand for 0.5 h, followed by vortexing for another 0.5 h to facilitate the adsorption of Stattic onto the PEG-MSNs.

The resulting precipitate, which consisted of PEG-MSNs loaded with Stattic, was collected by centrifugation at 12,000 rpm for 10 min. The precipitate was then washed with 600 µL of acetonitrile to remove any unbound Stattic. The Stattic-loaded PEG-MSNs were resuspended in acetonitrile, and the nanoparticles were separated from the drug by centrifugation at 12,000 rpm for 10 min. The supernatant, which contained the extracted Stattic, was filtered through a 0.45 µm membrane filter to remove any particulate matter. The filtered supernatant was then analyzed using high-performance liquid chromatography (HPLC) to determine the amount of Stattic loaded onto the PEG-MSNs.

To evaluate the release profile of the Stattic-loaded PEG-MSNs, the concentration of Stattic with the highest drug loading efficiency was determined as described above. The excess drug was removed by centrifugation at 12,000 rpm for 10 min, and the resulting precipitate was resuspended in PBS. The suspension was then stirred at room temperature to allow for the controlled release of Stattic from the PEG-MSNs. At various time intervals (0 h, 1 h, 2 h, 4 h, 6 h, 8 h, 12 h, and 24 h), 150 µL of the supernatant was collected and centrifuged at 12,000 rpm for 10 min to separate the released Stattic from the PEG-MSNs. An equal volume (150 µL) of PBS was added to each collected supernatant to maintain a consistent volume for analysis. The resulting samples were filtered through a 0.45 µm membrane filter and analyzed using HPLC to determine the amount of Stattic released from the PEG-MSNs at each time point.

### 3.5. In Vitro Cytotoxicity Assessment

The cytotoxicity and apoptotic effects of PEGylated mesoporous silica nanoparticles (PEG-MSNs) and PEG-MSNs loaded with the chemotherapeutic drug Stattic on RAW 264.7 cells, a murine macrophage line, were assessed using the MTT (3-(4,5-dimethylthiazol-2-yl)-2,5-diphenyltetrazolium bromide) assay and an apoptosis detection kit, respectively.

For the MTT assay, 1 × 10^4^ RAW 264.7 cells were seeded per well in a 96-well tissue culture plate and incubated at 37 °C in a humidified atmosphere containing 5% CO_2_ for 12 h. Next, the growth medium was removed, and the cells were exposed to fresh medium containing varying concentrations of PEG-MSNs and PEG-MSNs-Stattic. The cells were then incubated for an additional 24 h to allow for potential cytotoxic effects to manifest. After the incubation period, 10 µL of a 5 mg/mL MTT reagent solution was added to each well. MTT is a yellow tetrazolium salt that is reduced to purple formazan crystals by metabolically active cells, particularly those with active mitochondria. The cells were incubated with MTT reagent for 4 h, allowing for the formation of formazan crystals. The supernatant was then carefully removed, and 100 µL of dimethyl sulfoxide (DMSO) was added to each well to dissolve the blue-purple formazan crystals. The absorbance of the resulting solution at 570 nm was measured using a microplate reader (BioTek, Synergy H1, Winooski, VT, USA). The absorbance values obtained are proportional to the number of viable cells in each well and can be used to calculate the cell viability as a percentage relative to a control group treated with medium alone.

To assess the apoptotic effects of PEG-MSNs and PEG-MSNs-Stattic on RAW 264.7 cells, an apoptosis detection kit (Annexin V-FITC/PI, BBI, Cardiff, UK) was used. This kit utilizes the fluorescent dyes Annexin V-FITC (fluorescein isothiocyanate) and propidium iodide (PI) to distinguish between viable, early apoptotic, late apoptotic, and necrotic cells. Annexin V binds to phosphatidylserine, a phospholipid that is translocated from the inner to the outer leaflet of the plasma membrane during the early stages of apoptosis. In contrast, PI is a nucleic acid stain that penetrates cells with compromised plasma membranes, such as those in the late stages of apoptosis or necrosis. By analyzing the fluorescence emitted by cells stained with both dyes using a flow cytometer, the proportion of cells in each stage of apoptosis can be determined.

### 3.6. Establishment and Treatment of Tumor Models

To assess the therapeutic efficacy of PEGylated mesoporous silica nanoparticles (PEG-MSNs) loaded with the small-molecule drug Stattic and the combination of PEG-MSNs-Stattic with an anti-programmed cell death-ligand 1 (aPD-L1) antibody in immunotherapy, a series of tumor therapy experiments were conducted using a BALB/c mouse model of colon cancer. Prior to the experiments, the mice were acclimated to the laboratory environment for one week to minimize stress and allowed to adjust to their surroundings. Hair removal from the backs of the mice was performed to facilitate the injection of tumor cells and the monitoring of tumor growth. On day 1, a suspension containing 1 × 10^6^ CT26-Luc tumor cells, a luciferase-expressing colon cancer cell line, was inoculated subcutaneously into the right hindlimbs of the mice to establish the colon cancer model.

The mice were then randomly divided into five treatment groups: (1) UNTX (untreated control), (2) PEG-MSNs (PEGylated mesoporous silica nanoparticles alone), (3) Stattic (free Stattic without PEG-MSNs), (4) PEG-MSNs-Stattic (PEG-MSNs loaded with Stattic), and (5) PEG-MSNs-Stattic+aPD-L1 (combination of PEG-MSNs-Stattic and aPD-L1 antibody). When the average tumor size reached approximately 100 mm^3^, the mice in groups 2–5 were treated with Stattic, PEG-MSNs, or PEG-MSNs-Stattic via intratumoral injection at a dose of 2 mg/kg every 2 days. Additionally, the mice in group 5 were administered aPD-L1 antibody via intratumoral injection at a dose of 100 μg per tumor every 2 days.

The tumor volume and weight of the mice were measured, and the mice were observed every 2 days throughout the treatment period. Tumor volume was calculated using the following formula: length × width × width/2. This formula allows for the estimation of tumor size and provides a standardized method for monitoring tumor growth over time. To monitor the bioluminescence signal generated by the luciferase-expressing cancer cells, a small animal fluorescence imaging system was used every 5 days. Ten minutes after an intraperitoneal injection of d-luciferin potassium salt (Thermo Scientific Pierce, Waltham, MA, USA) at a concentration of 1.5 mg/mL and a dose of 10 μL/g, the mice were imaged. The exposure time for each imaging session was set to 1 min. Bioluminescence imaging provides a noninvasive method for assessing tumor burden and response to treatment by detecting the light emitted by luciferase-expressing tumor cells in response to a luciferin substrate.

### 3.7. Biodistribution of PEG-MSNs in Tumors

To investigate the biodistribution and tumor targeting ability of PEGylated mesoporous silica nanoparticles (PEG-MSNs) labeled with the near-infrared fluorescent dye NHS-Cy5.5, a series of in vitro and in vivo experiments were conducted.

First, 4 mg of PEG-MSNs was dissolved in 2 mL of deionized water, and 2 μL of NHS-Cy5.5 dye was added to the solution. NHS-Cy5.5 is a near-infrared fluorescent dye that can be covalently conjugated to amine groups present on the surface of PEG-MSNs, allowing for the detection of nanoparticle distribution in biological tissues. The solution was stirred overnight at 4 °C to facilitate the conjugation of the dye to the PEG-MSNs. After the incubation period, the PEG-MSNs-Cy5.5 conjugates were collected by centrifugation at 14,800 rpm for 30 min. The pellet was then washed twice with PBS to remove any unbound or excess dye. Finally, the pellet was resuspended in 2 mL of PBS to obtain a 2 mg/mL solution of PEG-MSNs-Cy5.5 in PBS.

To explore the biodistribution of PEG-MSNs-Cy5.5 in tumors, the labeled nanoparticles were intratumorally injected into untreated mice as a control group. The in vivo fluorescence signal intensity of the Cy5.5 dye was monitored over a period of 48 h after injection. At the end of the observation period, the tumors and major organs, including the heart, liver, spleen, lung, and kidney, were collected from the mice. The tissues were rinsed twice in PBS to remove any residual blood, which could interfere with the detection of the fluorescence signal. The fluorescence signal intensity of the tissues was then measured using an Intravital Fluorescence Imaging System after 15 s of exposure to the Cy5.5 fluorescence channel. This system allows noninvasive detection and quantification of the near-infrared fluorescence signal emitted by labeled nanoparticles in tissues.

In addition to whole-tissue imaging, tumor samples were further analyzed by flow cytometry and confocal microscopy to detect the Cy5.5 fluorescence signal in specific immune cell populations, such as macrophages, dendritic cells, and T cells. Flow cytometry is a technique that measures the fluorescence intensity of individual cells and can be used to quantify the presence of labeled nanoparticles within specific cell populations. Confocal microscopy, on the other hand, provides high-resolution images of the cellular distribution of the fluorescent signal within tissue sections. To identify and analyze the immune cell populations of interest, the following fluorochrome-labeled anti-mouse antibodies were used for staining: PE-CD3, FITC-F4/80; PE-CD45, FITC-CD11c. These antibodies allowed for the identification and characterization of immune cells associated with PEG-MSNs-Cy5.5 in the tumor microenvironment.

### 3.8. Immunofluorescence Analysis of Tumors

Tumor samples were embedded in an ultralow temperature environment of −80 °C. The sections were then sliced at the optimal cutting temperature using a cryostat at a thickness of 8–10 μm. Sections were fixed with 4% paraformaldehyde for 15 min, washed twice with phosphate buffer solution (PBS), blocked with 3% bovine serum albumin (BSA) for 10 min, and incubated overnight at 4 °C with APC-CD8, FITC-CD4, PE-CD11b, and APC-Gr-1 (1:200) in 0.1% Triton X-100 in 1% BSA buffer. The sections were removed, placed at room temperature for 30 min every other day, and then washed three times with PBS-T to remove unbound fluorescent dye. Finally, the sections were stained with 1 μg/mL DAPI for 10 min and washed once with PBS-T. The cells were observed with a confocal microscope (Zeiss LSM 800, Oberkochen, Germany), and the imaging parameters of the different groups were not different.

### 3.9. Flow Cytometry Analysis of Tumors

Mice were sacrificed on day 16 to weigh the tumors and analyze the tumor microenvironment. The relative proportions of T cells, including CD8^+^ T cells, CD4^+^ T cells, and MDSCs, were examined via flow cytometry analysis. After the mice were euthanized, the tumor samples were collected and homogenized to obtain single cells. The single-cell suspension was then filtered through 300-mesh gauze, washed and centrifuged with PBS, and resuspended in FACS buffer solution (PBS containing 3% BSA, Shanghai, China). The cell suspension was stained with flow cytometry antibodies at room temperature for 30 min and finally detected using flow cytometry (BD Accurit C6 Plus, Franklin Lakes, NJ, USA). The following fluorochrome-labeled anti-mouse antibodies were used for staining: (a) PE-CD3, FITC-CD45, APC-CD8, and Percp-CD4; (b) FITC-CD45, PE-CD11b, and APC-Gr-1.

## 4. Conclusions

**Figure 5:**
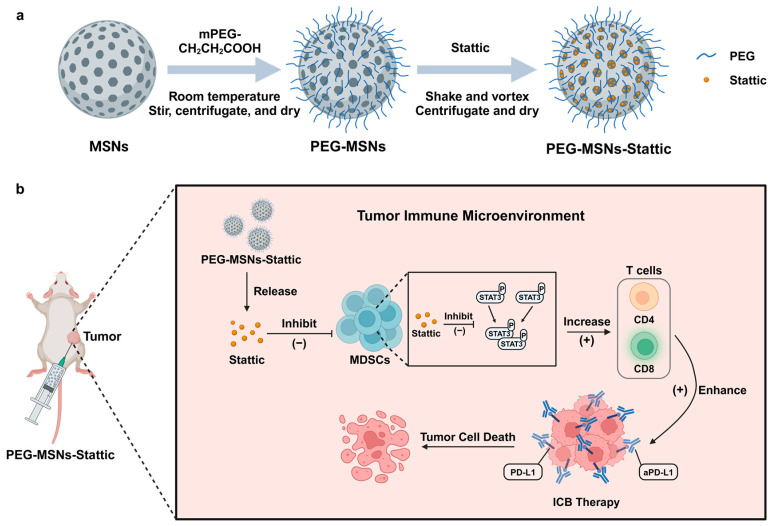
(**a**) Synthesis of PEG-MSNs-Stattic and (**b**) diagram of the use of PEG-MSNs-Stattic for enhancing antitumor efficacy.

In summary, our Stattic-loaded nanoparticles can achieve sustained drug release within tumors, reduce MDSC expansion, and increase T cell infiltration, which enhances the therapeutic effect of immune checkpoint antibodies (Figure 5). Considering that this delivery system is safe, simple to prepare, and inexpensive, we hope our approach can provide a general way to enhance antitumor immunotherapy.

## Figures and Tables

**Table 1:** The information of reagents and resources in this study.

Reagents & Resources	Source	Identifier
**Antibodies**		
FITC anti-mouse F4/80	Biolegend	Cat # 123108; RRID: AB_893502
PE anti-mouse CD45	Biolegend	Cat # 103106; RRID: AB_312971
FITC anti-mouse CD11c	Biolegend	Cat # 117306; RRID: AB_313775
PE anti-mouse CD3	Biolegend	Cat # 201412; RRID: AB_10804049
PE anti-mouse IFN-γ	Biolegend	Cat # 507806; RRID: AB_2830472
PE anti-mouse/human CD11b	Biolegend	Cat # 260042; RRID: AB_2536482
FITC anti-mouse CD45	Biolegend	Cat # 103108; RRID: AB_312973
APC anti-mouse Ly-6G/Ly-6C (Gr-1)	Biolegend	Cat # 108412; RRID: AB_313377
PE anti-mouse/human CD11b	Biolegend	Cat # 101208; RRID: AB_312791
Percp/Cyanine5.5 anti-mouse Ki-67	Biolegend	Cat # 652424; RRID: AB_2629531
APC anti-human CD8	Biolegend	Cat # 344722; RRID: AB_2829639
**Experimental Models: Cell Lines**		
RAW 264.7	Cell Bank of Shanghai Institutes for Biological Sciences	N/A
CT26-Luc	Cell Bank of Shanghai Institutes for Biological Sciences	N/A

## Data Availability

The data used to support the findings of this study are available from the corresponding author upon request.

## Supplementary Materials

The following supporting information can be downloaded at: https://www.mdpi.com/article/10.3390/molecules29112436/s1, Figure S1: Tumor growth curve of mice with UNTX and aPD-L1 treated groups. Data are shown as the means ± SEM. Statistical analysis was performed using Student’s *t*-test. ns, nonsignificant; Figure S2: Representative plots for the gating strategy of MDSCs in the tumor; Figure S3: Representative plots for the gating strategy of T cells in the tumor.
