# Supplementary material for: Drug-Loaded Mesoporous Silica Nanoparticles Enhance Antitumor Immunotherapy by Regulating MDSCs
Source: Molecules. 2024 May 22;29(11):2436. doi: 10.3390/molecules29112436 (PMC11173511; doi:10.3390/molecules29112436)
Supplement: Supplementary file 1 [file molecules-29-02436-s001.zip › molecules-2992951-supplementary.pdf]

Supplementary information for

Drug-loaded mesoporous silica nanoparticles enhance antitumor immunotherapy by regulating MDSCs

Changlin Xu†, Nida Amna†, Yuchen Shi†, Rong Sun, Chenhui Weng, Jiaoyu Chen, Huaxing Dai* and Chao Wang*

Laboratory for Biomaterial and Immunoengineering, Institute of Functional Nano & Soft Materials (FUNSOM), Jiangsu Key Laboratory for Carbon-based Functional Materials and Devices, Soochow University, Suzhou, Jiangsu 215123, China

*Corresponding author: Chao Wang: cwang@suda.edu.cn; Huaxing Dai: huaxingdai6@gmail.com

†Equally contribution

Supplementary information is available in online version of the paper.

Corresponding and requests for materials should be addressed to C.W.(cwang@suda.edu.cn).

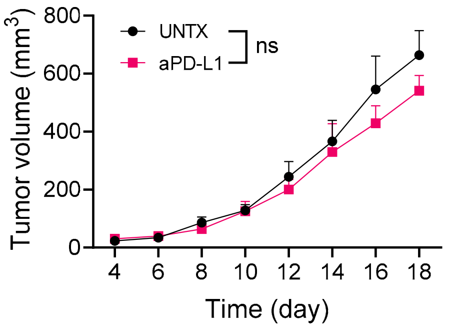

Figure S1. Tumor growth curve of mice with UNTX and aPD-L1 treated groups. Data are shown as the means ± SEM. Statistical analysis was performed using Student's t-test. ns, nonsignificant.

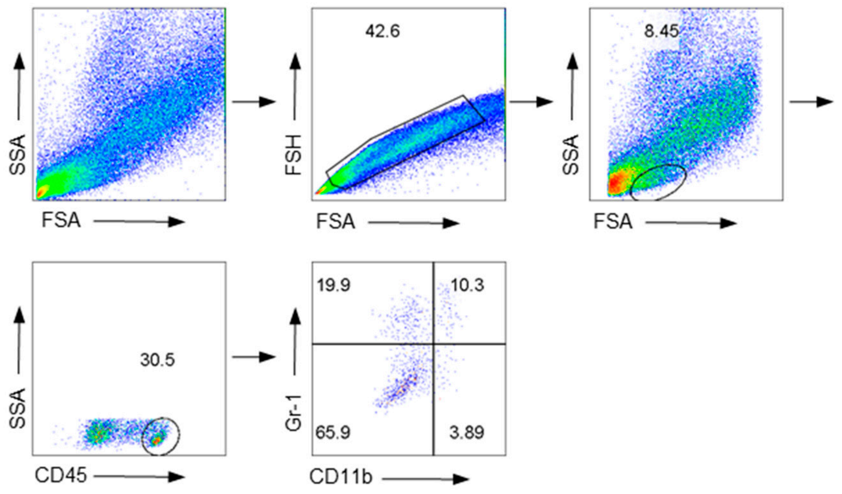

Figure S2. Representative plots for the gating strategy of MDSCs in the tumor.

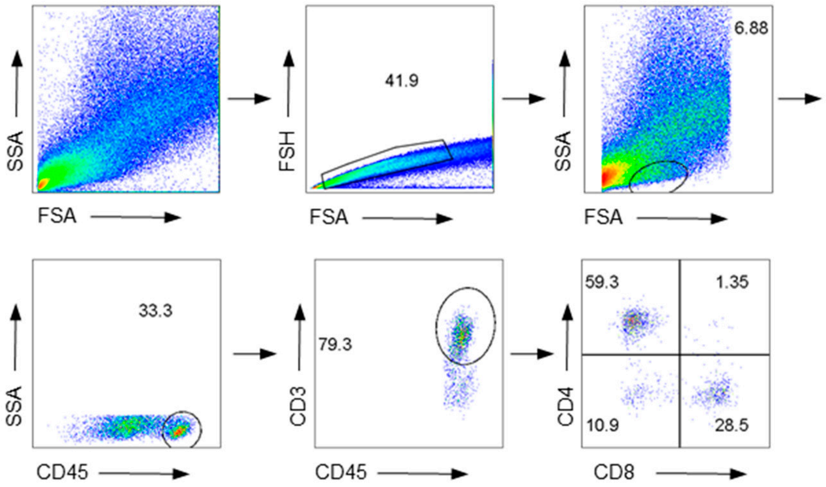

Figure S3. Representative plots for the gating strategy of T cells in the tumor.
